# Quantitative Evaluation of Low-Dose CT Image Quality Using Deep Learning Reconstruction: A Comparative Study of Philips Precise Image and GE TrueFidelity

**DOI:** 10.3390/jimaging11090317

**Published:** 2025-09-16

**Authors:** Jina Shim, Youngjin Lee, Kyuseok Kim

**Affiliations:** 1 Department of Radiotechnology, Wonkwang Health Science University, 514 Iksan-daero, Iksan-si 54538, Republic of Korea; eoeornfl@wu.ac.kr; 2 Department of Radiological Science, Gachon University, 191 Hambakmoe-ro, Yeonsu-gu, Incheon 21936, Republic of Korea; 3 Institute of Human Convergence Health Science, Gachon University, 191 Hambakmoe-ro, Yeonsu-gu, Incheon 21936, Republic of Korea

**Keywords:** deep learning image reconstruction, low-dose computed tomography, image quality assessment, quantitative phantom study, commercial CT image comparison

## Abstract

Reducing radiation exposure in CT imaging is critical, particularly in routine and repeat examinations. Deep learning image reconstruction (DLIR) has emerged as a key approach for maintaining diagnostic quality at low-dose acquisition settings. This study compared two DLIR algorithms of Philips Precise Image (PI) and GE TrueFidelity (TF) under an 80 kVp low-dose CT scenario, using the AAPM CIRS-610 phantom to replicate clinical imaging conditions. The phantom’s linearity, high-resolution, and artifact modules were scanned with Philips CT 5300 and GE Revolution CT scanners at low-dose parameters. Images were reconstructed using five DLIR presets, including PI (Smoother, Standard, Sharper) and TF (Middle, High), and evaluated with eight quantitative metrics, including SNR, CNR, nRMSE, PSNR, SSIM, FSIM, UQI, GMSD, and gradient magnitude. TF-High delivered the highest SNR (115.0–118.0 across modules), representing a 54–57% improvement over PI-Smoother, and achieved superior PSNR and the lowest GMSD, reflecting better preservation of structure in low-dose images. PI-Sharper provided the strongest gradient magnitude, emphasizing fine edge detail. Under low-dose CT conditions, TF-High offered the optimal balance of noise suppression and structure fidelity, while PI-Sharper highlighted fine detail enhancement. These findings show that DLIR settings must be tailored to clinical needs when operating under low-dose imaging protocols.

## 1. Introduction

Computed Tomography (CT) technology plays a pivotal role in modern medical diagnostic systems due to its capability for non-invasive cross-sectional image acquisition. However, ionizing radiation exposure to patients during examinations remains a major challenge in medical radiation safety management [1,2,3]. Particularly in chronic disease patient groups requiring long-term follow-up, or in pediatric and adolescent patient groups with relatively high radiosensitivity, the clinical application of the As Low As Reasonably Achievable (ALARA) principle, which dictates using the lowest possible radiation dose without compromising diagnostic information quality, has gained significant emphasis [4,5]. In response to these clinical demands, the development and introduction of Deep Learning-based Image Reconstruction (DLIR) technology in the field of image reconstruction are being evaluated as opening new horizons for CT image quality and radiation dose optimization [6,7,8].

The DLIR technology utilizes deep neural network models trained on vast amounts of image data. Numerous initial studies have reported that, compared to the conventional Filtered Back Projection (FBP) method or statistical Iterative Reconstruction (IR) techniques, DLIR offers various clinical advantages, such as effective control of image noise and improvement of inter-tissue contrast, even under significantly lower radiation dose conditions. Huang Wang et al. compared DLIR (DLIR-H) and ASIR-V40% in low-dose chest CT (80 kVp) and demonstrated that DLIR significantly reduced image noise and improved Contrast-to-Noise Ratio (CNR). It even achieved image quality comparable to 4% of standard-dose ASIR-V reconstructions, while preserving both anatomical and lesion detail [9]. Nayu Hamabuchi et al. evaluated DLIR in chest CT across standard, reduced, and ultra-low-dose protocols (down to ~0.8 mGy CTDIvol). DLIR delivered significantly higher Signal-to-Noise Ratio (SNR) compared to hybrid iterative reconstruction, and achieved superior lung texture evaluation and lesion detectability in patients with diverse pulmonary disease presentations [10]. These studies primarily focused on the outstanding noise-reduction capabilities of DLIR technology and the consequent potential for radiation dose reduction, suggesting the possibility of expanding the clinical application of low-dose CT protocols [11,12].

However, realizing the clinical potential of DLIR technology and optimally applying it in diverse diagnostic environments requires a thorough and objective comparative analysis of the detailed performance characteristics of DLIR algorithms developed and commercialized by different medical device manufacturers. To date, DLIR image quality assessment has tended to rely on subjective visual interpretation or qualitative grading by radiologists. Even when quantitative evaluation is attempted, the analysis has frequently been confined to some traditional metrics such as SNR or CNR [13,14,15]. Considering that image quality is not solely determined by noise levels but also influenced by the interplay of complex factors, including spatial resolution, fidelity of structural information, sharpness of boundaries, and potential generation of artifacts, evaluations based on limited metrics have inherent limitations in fully understanding the multifaceted performance of DLIR algorithms. Importantly, individual algorithms may excel in specific aspects of image quality while exhibiting unforeseen drawbacks in others [12,16].

Therefore, a comprehensive approach that applies diverse quantitative image-quality assessment factors to analyze the multidimensional characteristics of DLIR algorithms is necessary. The importance of such an approach is further highlighted in scenarios where low-kilovoltage peak (low-kVp) scanning techniques are used concomitantly with DLIR. Low-kVp scanning offers the distinct advantage of enhancing image contrast by augmenting X-ray attenuation differences between tissues; however, it can simultaneously lead to increased noise due to photon starvation and the potential degradation of spatial resolution [17,18]. Meticulous verification is required to ascertain whether DLIR technology can effectively compensate for these inherent drawbacks of low-kVp scanning and ensure the clinical validity of low-dose applications. Despite the growing clinical adoption of DLIR technologies, direct comparative studies systematically evaluating commercial algorithms, such as Philips’ Precise Image (PI) and GE Healthcare’s TrueFidelity (TF), using comprehensive quantitative metrics under identical low-kVp (80 kVp) and low-dose conditions remain scarce.

This study aims to address this research gap by applying the PI and TF reconstruction techniques to 80 kVp low-dose CT data and conducting a broad range of quantitative evaluations, including noise characteristics, signal fidelity, structural similarity, and edge characteristics. Such a multifaceted comparative evaluation can provide crucial scientific evidence for determining the potential performance and optimal applicability of each DLIR algorithm in specific clinical application areas, such as low-kVp and low-dose chest CT. To our knowledge, this is the first study to directly and systematically compare PI and GE TF deep learning reconstruction algorithms under identical low-dose (80 kVp) CT acquisition conditions using the AAPM CIRS-610 phantom. Unlike previous work that evaluated DLIR within single-vendor systems or using limited image quality metrics, our study employs a comprehensive suite of quantitative measures such as spanning noise suppression, structural fidelity, and edge sharpness to generate a multidimensional performance profile. This approach not only fills a critical gap in the literature but also provides clinically actionable insights, offering radiologists and technologists clearer guidance on selecting optimal DLIR settings for low-dose imaging scenarios. Therefore, this study’s specific objective is to conduct a detailed comparative evaluation of the image quality characteristics between PI and TF reconstruction techniques under 80 kVp low-dose phantom CT image conditions using various quantitative metrics and to elucidate their differences.

## 2. Materials and Methods

### 2.1. Quantitative Physical Phantom

This study utilized a standard phantom (Model CIRS-610, Nuclear Associates, Carle Place, NY, USA) designed by the American Association of Physicists in Medicine (AAPM) for the quantitative quality assessment of CT images [19]. This phantom is composed of multiple functions that allow the measurement of various image quality factors. For this research, three cross-sections obtained from the linearity, high-resolution, and artifact assessment modules were utilized for the analysis. The linearity assessment module contains inserts with different attenuation coefficients (Hounsfield Units, HU), making it suitable for comparing image quality under various HU conditions, particularly image signal characteristics and noise suppression capabilities. The high-resolution module includes structures that enable quantitative evaluation of the spatial resolution of fine structures, while the artifact module is used to examine quality changes under high-contrast image conditions, focusing on artifact patterns caused by high HU inserts.

### 2.2. CT Equipment and Scanning Parameters

Images were acquired using a CT 5300 (Philips Medical Systems, Cleveland, OH, USA) and Revolution CT (GE Healthcare, Waukesha, WI, USA). Both systems were scanned using standard- and low-dose protocols. To ensure reliable quantitative comparison between the two scanners, the protocols were configured by fixing the tube voltage and adjusting the tube current (mA) based on the same Computed Tomography Dose Index volume (CTDIvol). The standard-dose protocol for the CT 5300 system was configured at 120 kVp, 33 mA, and a CTDIvol of 2.42 mGy, while the low-dose protocol was set at 80 kVp, 19 mA, and a CTDIvol of 0.46 mGy. For Revolution CT, the standard-dose protocol was set at 120 kVp, 80 mA, and a CTDIvol of 2.55 mGy, which was an mA adjustment to achieve a CTDIvol similar to that of CT 5300. The low-dose protocol was set at 80 kVp, 40 mA, and a CTDIvol of 0.48 mGy, maintaining a dose level similar to the low-dose condition of the Philips scanner. Both scanners were set to helical scan mode, craniocaudal direction, 1 mm slice thickness and interval, and a 0.5 s rotation time. The pitch value was set to 0.8 for the Philips scanner and 0.992:1 for the Revolution CT. Table 1 summarizes the conditions. All scan conditions were maintained as consistently as possible to enhance the reliability of quantitative comparisons between the equipment.

### 2.3. Image Reconstruction

Raw data acquired at low doses were post-processed using DLIR techniques provided by each manufacturer. For CT 5300, the PI algorithm was applied and reconstructed at three strength levels: Smoother, Standard, and Sharper. PI is a deep-learning-based image reconstruction technique developed by Philips, designed to restore high-quality images, even from low-dose scans, by training a deep convolutional neural network using supervised learning. This algorithm aims to preserve the noise suppression performance of IR while maintaining the familiar image texture of FBP images. Neural network training is performed based on standard-dose CT data, which have a clinically desirable image appearance.

First, low-dose images that accurately reflect photon noise and electronic noise characteristics are generated through simulations based on high-dose data. To replicate low dose CT acquisition conditions, we simulated noise on high dose CT images acquired using the same CT scanner and acquisition protocol. The AAPM CIRS 610 phantom and patient datasets were first scanned at standard (reference) dose levels to provide baseline images. Because patient body mass index (BMI) strongly influences image noise, the dataset was stratified into normal, overweight, and obese groups to establish realistic noise levels for each condition. Here, this study was conducted in accordance with the principles of the Declaration of Helsinki and was approved by the Severance hospital Institutional Review Board (IRB No. 4-2024-0123). Low-dose conditions were modeled using the widely adopted Poisson–Gaussian noise mixture model [20], which reflects the physics of CT acquisition as Equation (1).

(1) $$y(x)=p(x)+\eta (p(x))\delta ,\;\eta^{2}(p(x))=\alpha p(x)+\sigma^{2}$$

where x denotes the pixel position, while y and p correspond to the degraded and ideal images, respectively, excluding any noise contribution. The function η(∙) indicates the standard deviation of the noise distribution, and δ represents zero-mean independent random noise with a standard deviation of one. The parameter *α* reflects the Poisson signal-dependent noise component, whereas σ represents the standard deviation of the Gaussian signal-dependent noise. Using this framework, *α* and σ were measured from the acquired CT images to quantify the level of noise according to patient BMI. In this model, the Poisson component represents the signal-dependent quantum noise, while the Gaussian term accounts for electronic and system-related noise. Noise parameters (*α* and *σ*) were quantified by analyzing homogeneous image patches (16 × 16 pixels) using a patch-based noise parameter estimation method. The parameters were derived from overweight and obese patient images, with measured *α* ≈ 0.15 ± 0.10 and *σ* ≈ 3.15 ± 1.25 for overweight and α ≈ 0.31 ± 0.07 and σ ≈ 4.16 ± 0.57 for obese patients. In setting the low-dose imaging conditions in Section 2.2, the noise parameter values in the images of CTDIvol, as well as overweight and obese patients, were considered. After acquiring images under various imaging conditions using the AAPM phantom, the low-dose CT dataset was obtained after selecting the low-dose imaging conditions, as shown in Table 1, in considering the degree of similarity between CTDIvol and the noise parameter values of actual patients. These low-dose images provided a controllable but clinically realistic dataset for evaluating the performance of PI and TF algorithms under low-dose conditions. These images are designed to faithfully reflect noise properties under real-world low-dose conditions.

**Figure 1:**
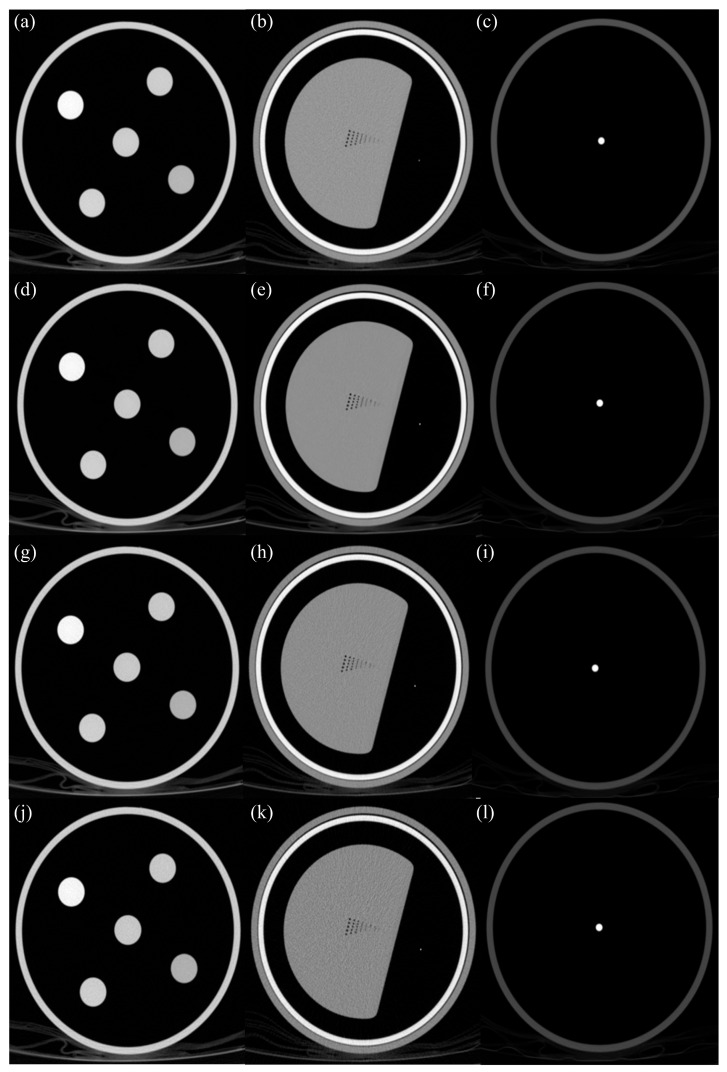
Representative axial computed tomography (CT) images of the American Association of Physicists in Medicine (AAPM) phantom acquired with the Philips scanner, demonstrating the effects of different reconstruction algorithms. Images in the first row (**a**–**c**) are reconstructed using the standard Filtered Back Projection (FBP) as a reference. Images in the subsequent rows are low-dose acquisitions reconstructed with three different strength levels of the Precise Image (PI) algorithm: PI-Smoother (**d**–**f**), PI-Standard (**g**–**i**), and PI-Sharper (**j**–**l**). Columns from left to right represent the linearity module (**a**,**d**,**g**,**j**), the high-resolution module (**b**,**e**,**h**,**k**), and the artifact module (**c**,**f**,**i**,**l**), respectively.

Subsequently, the high-dose images are reconstructed using the FBP algorithm, and the neural network is trained to restore these FBP-based high-dose images from low-dose inputs. This training strategy considers various factors, such as the boundaries of fine structures in the image, tissue contrast, and noise texture, enabling the network to possess high robustness across diverse patient conditions (e.g., physique, anatomical structure, and radiation dose). Consequently, PI can achieve high-speed image reconstruction that reliably removes noise and ensures structural accuracy even at low doses, while maintaining image quality similar to that of traditional FBP images [21]. Figure 1 shows the representative images from the Philips scanner illustrating the standard FBP and different PI levels (Smoother, Standard, Sharper) across the phantom. For reproducibility, the computational environment was specified. Precise Image reconstructions were performed on a workstation equipped with a dual-CPU Intel Xeon Gold 4214 processor (2.2 GHz, 12 cores), an NVIDIA Quadro P620 (2 GB) and NVIDIA Quadro RTX A2000 (12 GB) GPU, 64 GB of main memory, and a 4.4 TB SSD (2.2 TB × 2). TrueFidelity reconstructions were performed on a Lenovo P520 workstation equipped with an Intel Xeon W-2145 CPU (3.7 GHz, 8 cores/16 threads), 96 GB DDR4 ECC memory (4×8 GB and 4×16 GB), and an NVIDIA Quadro P620 GPU (2 GB, VBIOS Gen 1: 86.07.71.00.01 or 86.07.8D.00.02; Gen 2: 86.07.90.00.A7).

For Revolution CT, TrueFidelity (TF), a deep-learning-based image reconstruction technique from GE Healthcare, was applied [22]. TF is a reconstruction engine based on a deep neural network (DNN) developed to overcome the physical modeling limitations of IR, aiming to restore image quality similar to that of high-dose FBP images, even from low-dose CT images. The TF training process is based on supervised learning. First, data acquired under high-dose conditions are reconstructed using the FBP method to generate ground-truth images. Corresponding low-dose images are constructed using clinical and phantom data acquired under various body types, anatomical regions, and radiation dose conditions. Based on these input-ground truth pairs, the DNN is trained to predict the ground truth images from the low-dose images. Learning is accomplished by calculating the loss value between the output and the ground truth images and iteratively optimizing millions of parameters through a backpropagation algorithm [23]. A sufficiently trained DNN processes sinogram data in real-time after scanning to generate TF images. This algorithm can be applied at three levels—Low, Medium, and High—according to the image’s noise reduction strength, and can be flexibly selected according to clinical purposes and user preferences. As a result, TF provides improved noise suppression performance and diagnostic quality while maintaining the spatial resolution and fine structure preservation capabilities of the image compared to IR.

**Figure 2:**
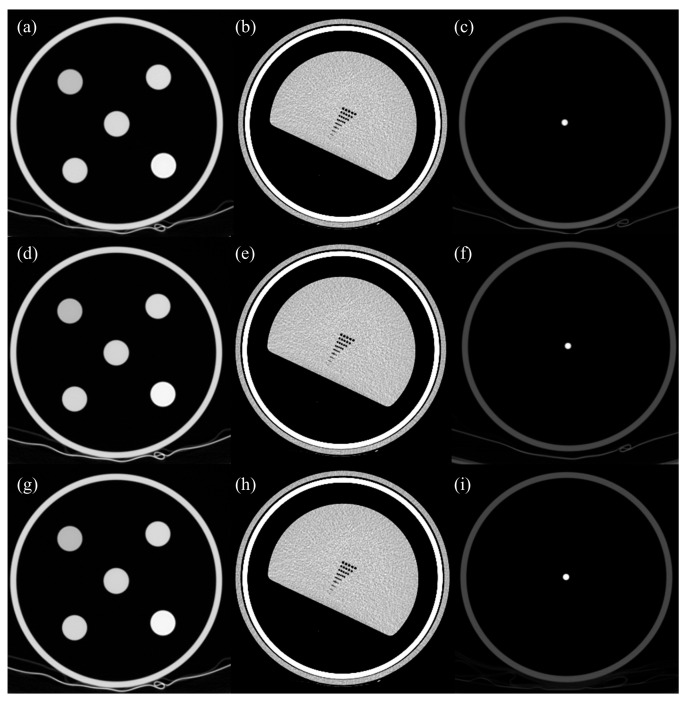
Representative axial CT images of the AAPM phantom acquired with the GE Revolution scanner, illustrating the effects of different reconstruction algorithms. Images in the first row (**a**–**c**) are reconstructed using the standard Filtered Back Projection (FBP) as a reference. Images in the subsequent rows are low-dose acquisitions reconstructed with two different strength levels of the TF algorithm: TF-Middle (TF-M, Level 1) (**d**–**f**) and TF-High (TF-H, Level 2) (**g**–**i**). Columns from left to right represent the linearity module (**a**,**d**,**g**), the high-resolution module (**b**,**e**,**h**), and the artifact module (**c**,**f**,**i**), respectively.

Figure 2 illustrates the three reconstruction modes applied to the AAPM phantom: standard filtered back projection (FBP), TF-Middle (TF-M), and TF-High (TF-H). The FBP image serves as a conventional baseline, while TF-M and TF-H represent two levels of GE’s deep learning-based reconstruction algorithm. Compared to FBP, TF-M shows a marked reduction in image noise while maintaining anatomical edge sharpness, particularly in linearity and artifacts. TF-H further enhances noise suppression and structural uniformity, yielding visually smoother textures and improved contrast homogeneity, particularly in low-contrast regions of the phantom. These visual differences are consistent with the underlying design of TF, which utilizes a supervised DNN trained on paired high- and low-dose image datasets. As described previously, TF aims to replicate high-dose image quality by learning to minimize the error between low-dose input and high-dose ground-truth images. The level-dependent output observed in Figure 2 reflects the algorithm’s ability to adapt to different clinical imaging requirements through variable noise reduction strengths.

**Figure 3:**
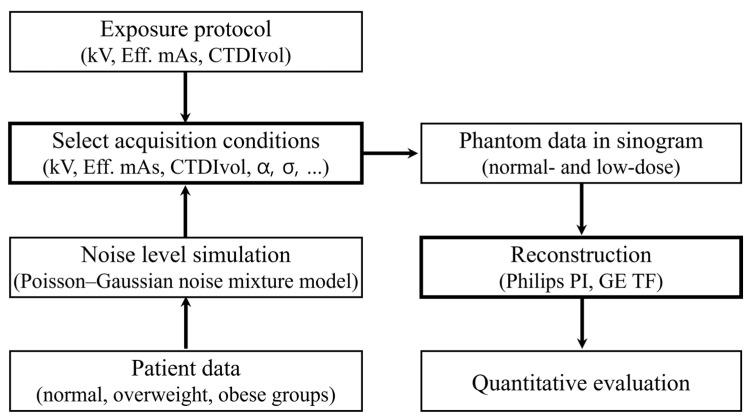
Simplified flowchart of quantitative evaluation of low-dose CT image quality using deep learning reconstruction.

In summary, the definition of low-dose CT conditions was guided by both phantom and real patient datasets. Specifically, we compared the CTDIvol and noise parameter values obtained from overweight and obese patients with those derived from phantom acquisitions. By aligning the phantom acquisition settings with the noise characteristics observed in patient images, we ensured that the simulated low-dose conditions faithfully reflected realistic clinical scenarios. Based on this criterion, we obtained paired phantom datasets at normal-dose and low-dose levels. Subsequently, both datasets were reconstructed using two DLIR algorithms (Philips PI and GE TF), and image quality was evaluated across multiple quantitative metrics, including SNR, CNR, SSIM, FSIM, and GMSD. This design allows our phantom study not only to provide controlled and reproducible comparisons but also to remain anchored in clinically realistic noise characteristics. For clarity, the overall workflow is summarized in a schematic flow diagram of Figure 3.

### 2.4. Quantitative Image Quality Assessment

Quantitative image quality assessment was performed by comparing images reconstructed using DLIR techniques against the baseline FBP-reconstructed images from the respective scanners. To systematically categorize the evaluation items, they were categorized into three areas according to the analysis objectives of the research results. First, the ‘Noise and Signal Fidelity’ category, which assesses the fidelity of the signal within the image and noise suppression capability, included SNR, Peak SNR (PSNR), and normalized Root Mean Squared Error (nRMSE) [24,25,26]. Second, the ‘Structural Similarity Assessment’ category, evaluating the structural preservation capability between images, included the Structural Similarity Index (SSIM), Feature Similarity Index (FSIM), and Universal Quality Index (UQI) [27,28,29]. Third, the Edge Sharpness and Structural Distortion Assessment category, which analyzes the sharpness of image boundaries and fine structure preservation capability, was conducted through Global Mean Structural Dissimilarity (GMSD) [30] and Gradient Magnitude (GM). This classification of assessment metrics was applied in a structured manner from the research design stage to ensure consistency in the interpretation of each image quality element and clarity in the quantitative comparisons.

The SNR quantifies the quality of an image or data by converting the ratio between the energy of the effective signal component and the background noise to a logarithmic scale. When the average intensity of the signal is M and the standard deviation of the noise is β, the following equation (Equation (2)) defines the metric:

(2) $$SNR=10\times \log_{10}(\frac{M^{2}}{\beta^{2}})$$

The PSNR is a quantitative quality metric that represents the degree of distortion between a reference image and a comparative image on a logarithmic scale; a higher value indicates superior image reproduction quality. When the maximum brightness value of the image is L and the mean squared error between the two images is E, PSNR is defined as follows in Equation (3):

(3) $$PSNR=20\times \log_{10}(\frac{L}{\sqrt{E}}),\;E=\;\frac{1}{M\times N}\sum_{i=1}^{M}\sum_{j=1}^{N}{\left(A\left(i,j\right)-B\left(i,j\right)\right)}^{2}$$

Here, E is the value obtained by squaring the difference in pixel values between the reference image A and comparative image B and obtaining their average. $A\left(i,j\right)$ represents the pixel value at position $\left(i,j\right)$ of the original image, $B\left(i,j\right)$ represents the pixel value at the same position in the processed image, and M and N represent the vertical and horizontal resolutions of the image, respectively. Therefore, M×N signifies the total number of pixels.

nRMSE is the normalized value of RMSE and represents the average magnitude of the prediction error in the same units as the original by taking the square root after averaging the squared differences between the predicted and actual observed values. When the difference between the model’s predicted value and the actual value is $e_{i}$ and the total number of observed values is n, RMSE is defined as follows in Equation (4):

(4) $$RMSE=\;\sqrt{\frac{1}{n}\sum_{i=1}^{n}e_{i}^{2}},\;e_{i}=\;T_{i}-\;P_{i}$$

Here, the error is defined as the difference between each observed value $T_{i}$ and predicted value $P_{i}$. Since nRMSE is based on squared errors, it reacts more sensitively to large errors and is a suitable performance indicator for datasets assumed to have a normal distribution. nRMSE is widely used, particularly for evaluating how close, on average, predicted values are to actual values. To address differences in intensity scale across imaging systems and to ensure fair comparison, RMSE values are often normalized. In this study, we calculated normalized nRMSE by dividing the RMSE by the dynamic range of the data. This allows for consistent evaluation regardless of varying intensity distributions between reconstruction methods or vendors.

CNR quantifies the ability to distinguish a lesion from the background by comparing the contrast between the two regions against their respective noise levels. A higher CNR indicates clearer differentiation between the lesion and background, suggesting improved visibility. When the average signal intensity of the lesion is denoted as $I_{t}$, the background intensity as $I_{b}$, and the noise levels of the lesion and background as $\sigma_{a}$ and $\sigma_{b}$ respectively, CNR is defined as follows in Equation (5):

(5) $$CNR=\;\frac{\left|I_{t}-I_{b}\right|}{\sqrt{\sigma_{t}^{2}+\sigma_{b}^{2}}}$$

GMSD is an indicator that quantifies the overall quality of an image through the standard deviation of the similarity map after calculating the gradient magnitude similarity between a reference image and a distorted image. Let the gradient magnitudes of the original image X and comparative image Y be $G_{X}\left(i\right)$ and $G_{Y}\left(i\right)$ respectively. From these, the similarity map $S\left(i\right)$ is defined as follows in Equation (6):

(6) $$S\left(i\right)=\;\frac{2G_{X}\left(i\right)G_{Y}\left(i\right)+\varepsilon }{G_{X}{\left(i\right)}^{2}+G_{Y}{\left(i\right)}^{2}+\varepsilon }$$

Here, ε is a small positive constant for numerical stability. This similarity is calculated for each pixel, and the closer $S\left(i\right)$ is to 1, the higher the structural similarity between the two images. Subsequently, the overall image quality score is calculated as the standard deviation of the similarity map $S\left(i\right)$, which defines the GMSD metric in Equation (7):

(7) $$GMSD=\;\sqrt{\frac{1}{N}\overset{N}{\underset{i=1}{\sum }}{\left(S\left(i\right)-\bar{S}\right)}^{2}}$$

Here $\bar{S}$ is the mean of $S\left(i\right)$ and N is the total number of pixels. GMSD reflects the spatial distribution of distortion, and a lower value indicates higher quality. This is because GMSD sensitively reflects the non-uniformity of local structural damage within the image.

SSIM is a metric that measures the structural similarity between two images, designed based on how the human visual system perceives luminance, contrast, and structural information. For a reference image R and a comparative image S, let the luminance means be $\mu_{R}$ and $\mu_{S}$ respectively, variances be $\sigma_{R}^{2}$ and $\sigma_{S}^{2}$, and covariance be $\sigma_{RS}$. SSIM is then defined as follows in Equation (8):

(8) $$SSIM\left(R,S\right)=\;\frac{\left(2\mu_{R}\mu_{S}+c_{1}\right)\left(2\sigma_{RS}+c_{2}\right)}{\left(\mu_{R}^{2}+\mu_{S}^{2}+c_{1}\right)\left(\sigma_{R}^{2}+\sigma_{S}^{2}+c_{2}\right)}$$

Here, $c_{1}$ and $c_{2}$ are small constants used to prevent numerical instability, which can occur when the luminance or contrast values are close to zero. Typically, they are set as $c_{1}=\;{\left(k_{1}L\right)}^{2}$ and $c_{2}=\;{\left(k_{2}L\right)}^{2}$, where L is the maximum range of the pixel values. The closer SSIM is to 1, the higher the similarity. In image quality assessment, SSIM, unlike PSNR or nRMSE, reacts sensitively to structural distortions, providing results that are more consistent with perceptual quality judgment.

FSIM is an image quality assessment metric based on the principle that the human visual system primarily responds to low-level features rather than to structures when perceiving an image. FSIM particularly utilizes Phase Congruency (PC) and GM as primary and auxiliary features, respectively, and calculates the similarity between these two features to derive a comprehensive image similarity. Let the PCs at a location i in the reference and comparative images be $P_{1}\left(i\right)$ and $P_{2}\left(i\right)$, respectively, and the gradient magnitudes be $G_{1}\left(i\right)$ and $G_{2}\left(i\right)$. The similarity of each feature is defined as follows in Equation (9):

(9) $$S_{P}\left(i\right)=\;\frac{2P_{1}\left(i\right)P_{2}\left(i\right)+\varepsilon_{1}}{P_{1}{\left(i\right)}^{2}+P_{2}{\left(i\right)}^{2}+\varepsilon_{1}},\;\;S_{G}\left(i\right)=\;\frac{2G_{1}\left(i\right)G_{2}\left(i\right)+\varepsilon_{2}}{G_{1}{\left(i\right)}^{2}+G_{2}{\left(i\right)}^{2}+\varepsilon_{2}}$$

Finally, FSIM is calculated by averaging the combined result of these two similarities, weighted by PC in Equation (10):

(10) $$FSIM=\;\frac{\sum_{i\epsilon \Omega }max\left(P_{1}\left(i\right),\;P_{2}\left(i\right)\right)\times \left(S_{P}\left(i\right)\times S_{G}\left(i\right)\right)}{\sum_{i\epsilon \Omega }max\left(P_{1}\left(i\right),\;P_{2}\left(i\right)\right)}$$

Here, Ω is the entire pixel domain, and $\varepsilon_{1}$, $\varepsilon_{2}$ are small positive constants for numerical stability. An FSIM value close to 1 indicates that the two images are visually similar.

UQI is a quality assessment metric that expresses the similarity between two images in terms of luminance, contrast, and structure as a single scalar value. Given a reference image X and a comparison image Y, let $\bar{x}$, $\bar{y}$ denote their respective means, $\sigma_{x}^{2}$, $\sigma_{y}^{2}$ their variances, and $\sigma_{xy}$ their covariance. Equation (11) defines UQI as:

(11) $$UQI\left(X,Y\right)=(\frac{2\bar{xy}}{{\bar{x}}^{2}+{\bar{y}}^{2}})\times (\frac{2\sigma_{xy}}{\sigma_{x}^{2}+\sigma_{y}^{2}})$$

This is composed of two multiplicative terms. The first term reflects the luminance similarity, which approaches 1 as the average intensities of the two images become more similar. The second term captures the structural similarity by incorporating both the variance and covariance, reflecting the degree of shape similarity between the two images.

GM is a useful characteristic for detecting loc1al structural changes within an image and is an indicator that emphasizes regions where brightness changes abruptly (e.g., boundaries and contours). For an image F, derivatives are calculated using a horizontal mask $H_{x}$ and a vertical mask $H_{y}$, and GM is defined as follows based on these in Equation (12):

(12) $$GM={\left(F⊛H_{x}\right)}^{2}\left(i,j\right)+{\left(F⊛H_{y}\right)}^{2}*\left(i,j\right)$$

All aforementioned image quality metrics were calculated using MATLAB (MathWorks Corp., Natick, MA, USA, R2022a). Slices corresponding to each of the three sections of the phantom module were analyzed to quantitatively compare the multifaceted performance among the image reconstruction techniques. To ensure reproducibility, three independent acquisitions were performed for each condition, and the average values of these measurements were used for subsequent comparisons.

## 3. Results

### 3.1. Noise and Signa

#### 3.1.1. Linearity

**Figure 4:**
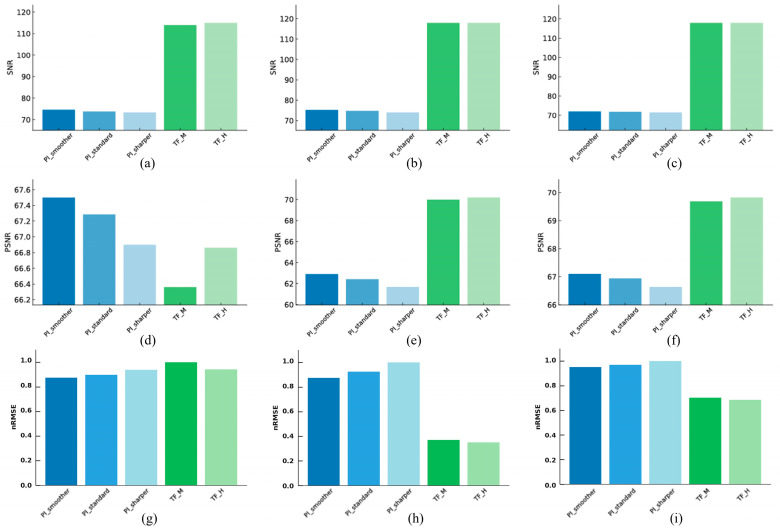
Quantitative comparison of noise and signal fidelity metrics for images reconstructed with Precise Image (PI-Smoother, PI-Standard, PI-Sharper) and TrueFidelity (TF-M, TF-H) algorithms under low-dose conditions. The first row shows the Signal-to-Noise Ratio (SNR) for the (**a**) linearity module, (**b**) high-resolution module, and (**c**) artifact module. The second row displays Peak Signal-to-Noise Ratio (PSNR) for the (**d**) linearity, (**e**) high-resolution, and (**f**) artifact. The third row presents normalized Root Mean Squared Error (nRMSE) for the (**g**) linearity, (**h**) high-resolution, and (**i**) artifact.

**Figure 5:**
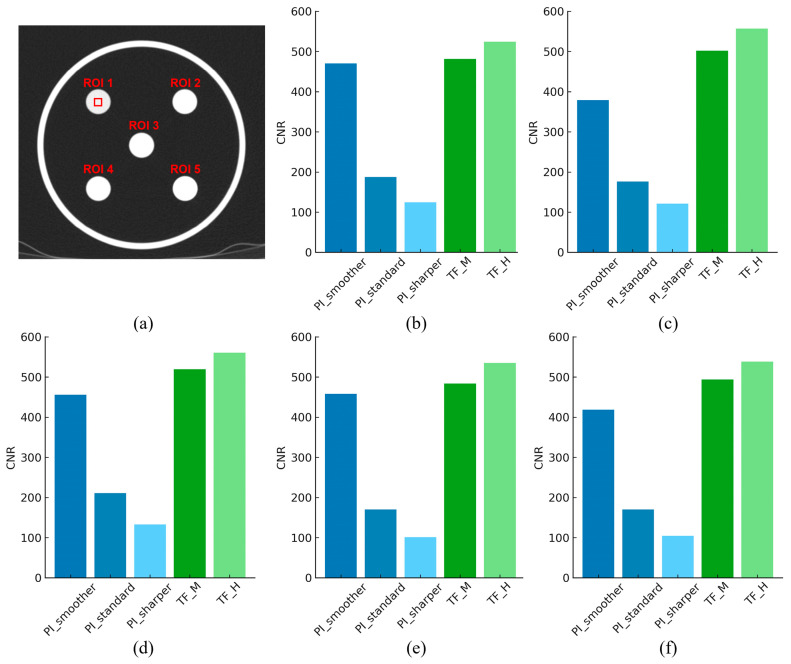
Representative axial computed tomography (CT) image of the American Association of Physicists in Medicine (AAPM) phantom acquired with the GE Revolution scanner, showing the regions of interest (ROIs), indicated by the red box, defined within the linearity module for contrast-to-noise ratio (CNR) measurement (**a**). Bar graphs (**b**–**f**) display the CNR values for each ROI (from ROI 1 to ROI 5) reconstructed using three strength levels of the Precise Image (PI) algorithm (PI_Smoother, PI_Standard, PI_Sharper) and two strength levels of the TrueFidelity (TF) algorithm (TF_M, TF_H).

Figure 4 summarizes the quantitative results for noise and signal fidelity metrics (SNR, PSNR, and nRMSE) across the different reconstruction algorithms and phantoms. PI-Smoother showed the best values among the PI settings for all three metrics: SNR 74.65, PSNR 67.50, and nRMSE 0.877. TF-H exhibited an SNR value of 115.0, a PSNR value of 66.86, and an nRMSE value of 0.944, which were superior to TF-M. Comparing the two algorithms, TF-H showed an approximately 54.1% higher SNR, indicating a superior noise suppression capability. The PSNR for TF-H was 0.64 lower, suggesting a similar level of signal preservation to that of PI-Smoother. nRMSE was 7.6% higher for TF-H. CNR values were measured by placing ROIs on five different materials within the linearity module, as illustrated in Figure 5. The ROIs, each sized 100×100 pixels, were positioned at the center of the circular regions corresponding to each material within the phantom to ensure consistency. PI-Smoother showed the best CNR values among the PI settings across all ROIs, with values of 470.64, 457.56, 455.64, 418.32, and 379.21, respectively. TF-H exhibited CNR values of 560.03, 557.39, 538.46, 535.25, and 524.22, which were superior to those of TF-M across all ROIs. Comparing the two algorithms, TF-H showed an approximately 25% higher average CNR, indicating improved lesion detectability.

#### 3.1.2. High-Resolution

PI-Smoother showed the best values among the PI settings for all three metrics: SNR 75.30, PSNR 62.90, and nRMSE 0.869. TF-H exhibited an SNR value of 118.0, a PSNR value of 70.19, and an nRMSE value of 0.375, which were superior to TF-M. Comparing the two algorithms, TF-H had an SNR approximately 56.7% higher, a PSNR approximately 13.5% higher, and an nRMSE approximately 56.8% lower than PI-Smoother, demonstrating excellent results in both noise suppression and signal fidelity.

#### 3.1.3. Artifact

PI-Smoother showed the best values among the PI settings for all three metrics: SNR 72.05, PSNR 67.09, and nRMSE 0.948. TF-H exhibited an SNR value of 118.0, a PSNR value of 69.82, and an nRMSE value of 0.693, which were superior to TF-M. Comparing the two algorithms, TF-H had an SNR approximately 63.8% higher and a PSNR approximately 4.1% higher than PI-Smoother. The nRMSE for TF-H was also approximately 27% lower, proving its superiority in both noise suppression and signal preservation.

### 3.2. Structural Similarity

#### 3.2.1. Linearity

**Figure 6:**
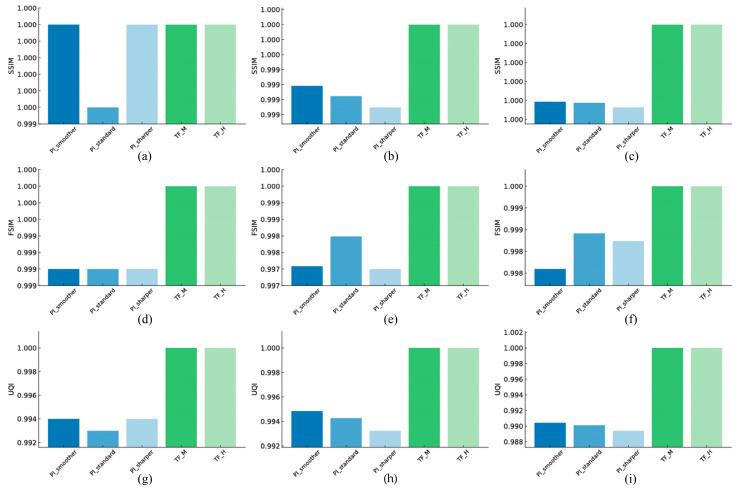
Quantitative comparison of structural similarity metrics for images reconstructed with Precise Image (PI-Smoother, PI-Standard, PI-Sharper) and TrueFidelity (TF-M, TF-H) algorithms under low-dose conditions. The first row shows the Structural Similarity Index Measure (SSIM) for (**a**) linearity, (**b**) high-resolution, and (**c**) artifact. The second row displays Feature Similarity Index Measure (FSIM) for (**d**) linearity, (**e**) high-resolution, and (**f**) artifact. The third row presents Universal Quality Index (UQI) for (**g**) linearity, (**h**) high-resolution, and (**i**) artifact.

Figure 6 illustrates the comparative structural similarity assessments using SSIM, FSIM, and UQI for the evaluated reconstruction techniques. PI-Smoother recorded an SSIM of 1.000, FSIM of 0.999, and UQI of 0.994, while TF-H showed perfect agreement, with a value of 1.000 for all three metrics. TF-H was approximately 0.1% higher in FSIM and approximately 0.6% higher in UQI.

#### 3.2.2. High-Resolution

PI-Smoother recorded an SSIM value of 0.9991, FSIM value of 0.9975, and UQI value of 0.9948. TF-H scored 1.000 on all metrics, being approximately 0.25% higher in FSIM and approximately 0.5% higher in UQI.

#### 3.2.3. Artifact

The PI-Smoother was measured at SSIM = 0.9996, FSIM = 0.9980, and UQI = 0.9904. TF-H also showed 1.000 for these, being approximately 0.2% higher in FSIM, and approximately 0.9% higher in UQI.

### 3.3. Edge Sharpness and Fine Structure Preservation

#### 3.3.1. Linearity

**Figure 7:**
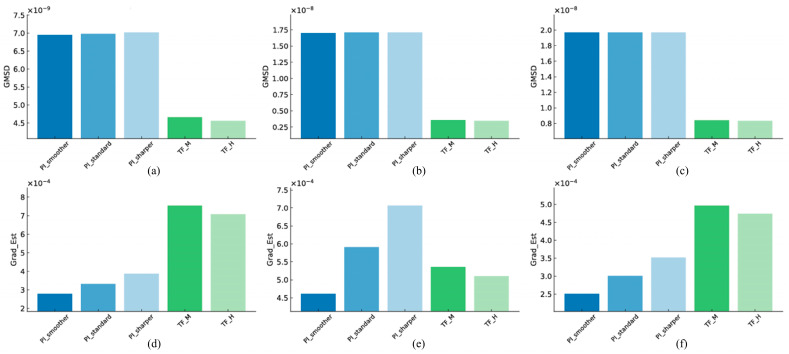
Quantitative comparison of edge sharpness and fine structure preservation metrics for images reconstructed with Precise Image and TrueFidelity algorithms under low-dose conditions. The top row shows Global Mean Structural Dissimilarity (GMSD) for (**a**) linearity, (**b**) high-resolution, and (**c**) artifact. The bottom row displays Gradient Magnitude for (**d**) linearity, (**e**) high-resolution, and (**f**) artifact.

Figure 7 shows the evaluation of edge sharpness using the Gradient and structural distortion using GMSD. Among the three PI levels, the Sharper level recorded the highest Gradient value at 3.87 × 10^−4^. Among the two TF levels, the Middle was the best at 7.55 × 10^−4^. Comparing the two reconstruction methods, TF-M showed an approximately 95.1% higher Gradient value than PI-Sharper, indicating superior edge delineation. Among the three PI levels for GMSD, Smoother showed the lowest value at 6.95 × 10^−9^. Among the two TF levels, High recorded the best result at 4.56 × 10^−9^. Comparing the results, TF-H had an approximately 34.4% lower GMSD than PI-Smoother, indicating a distinct advantage in terms of structural distortion suppression.

#### 3.3.2. High-Resolution

Among the three PI levels, Sharper showed the highest Gradient value at 7.07 × 10^−4^. Among the two TF levels, Middle was the best at 5.36 × 10^−4^. Comparing the two methods, PI-Sharper showed an approximately 31.9% higher value than TF-M, indicating superiority in terms of edge sharpness. Among the three PI levels for GMSD, PI-Smoother was the lowest at 1.70 × 10^−8^. Among the two TF levels, high was the best at 3.48 × 10^−9^. Comparing the results, TF-H had an approximately 79.5% lower GMSD than PI-Smoother, indicating significantly superior structural preservation performance.

#### 3.3.3. Artifact

Among the three PI levels, Sharper was the best for Gradient at 3.52 × 10^−4^. Among the two TF levels, TF-M showed the highest value at 4.97 × 10^−4^. Comparing the two reconstruction methods, TF-M showed an approximately 41.2% higher value than PI-Sharper, demonstrating superiority in terms of edge sharpness. Among the three PI levels for GMSD, Smoother was the lowest at 1.97 × 10^−8^. Among the two TF levels, TF-H was the best at 8.34 m × 10^−9^. Comparing the two methods, TF-H was approximately 57.6% lower than PI-Smoother, confirming its superior structural distortion suppression performance.

## 4. Discussion

This study aimed to compare and evaluate the quantitative image quality characteristics of PI and TF, two deep learning based CT image reconstruction techniques commercialized under 80 kVp low-dose conditions, using the AAPM phantom to consider the low-dose imaging environment of real patients. With advancements in CT technology, particularly the increasing importance of low-dose scanning techniques, DLIR technology is gaining attention as part of efforts to acquire diagnostically valuable images while minimizing radiation exposure [6,7]. This study objectively identifies the unique characteristics, advantages, and limitations of these DLIR algorithms under specific low-kVp conditions by analyzing their performance using multifaceted quantitative metrics. The results of this study provide specific information on the performance differences between PI and TF techniques in various aspects, such as noise reduction, structural preservation, and edge sharpness, which are considered valuable foundational data for selecting the optimal reconstruction technique and level for specific diagnostic purposes in future clinical settings.

In the noise and signal fidelity domains, the TF-H level generally showed a clear superiority over PI-Smoother in terms of SNR and nRMSE. Across the linearity, high-resolution, and artifact modules, TF-H recorded SNR values approximately 54–64% higher, demonstrating excellent noise suppression capability even in a low-dose environment. For PSNR, PI-Smoother was slightly superior or at a similar level only in the linearity module. However, in the high-resolution and artifact modules, TF-H recorded approximately 13.5% and 4.1% higher values, respectively, indicating overall superiority in signal fidelity as well. This implies that the TF algorithm can effectively achieve a balance between noise control and signal preservation, even in low-dose situations.

Regarding structural similarity, TF-H recorded nearly perfect scores in SSIM, FSIM, and UQI metrics, showing consistent structural concordance with the original image. Particularly in the high-resolution and artifact modules, FSIM and UQI values were up to 0.25% and 0.9% higher, respectively, compared to PI-Smoother, supporting TF’s superiority in fine structure preservation. This suggests that the TF algorithm also possesses strengths in maintaining the overall structural consistency of the image.

More detailed differences were observed in the comparison of edge sharpness and structural distortion suppression performance. Regarding the Gradient, PI-Sharper showed an approximately 31.9% higher value than TF-M in the high-resolution module, indicating sharper edge delineation. However, in the linearity and artifact modules, TF-M recorded approximately 95.1% and 41.2% higher values, respectively, indicating superior edge sharpness. This suggests that while PI-Sharper may be advantageous for sharply rendering fine structures in a high-resolution environment, TF-M may provide more consistent sharpness in depicting general tissue boundaries. In the GMSD metric, TF-H recorded the lowest values in all modules, being approximately 34.4% lower in the linearity module, approximately 79.5% lower in the high-resolution module, and approximately 57.6% lower in the artifact module, compared to PI-Smoother. These results demonstrate that TF-H is generally the most superior in terms of original structure preservation, suggesting that it could be an optimal choice for reliably reproducing images without fine structural distortion, especially in low-dose environments.

These results demonstrate that the PI and TF algorithms exhibit unique image reconstruction characteristics. While PI-Sharper excels in edge delineation in high-resolution environments, PI-Smoother is advantageous for overall noise suppression and structural distortion suppression. TF, particularly through its High and Middle levels, characteristically provides consistent and balanced image quality in terms of overall structural preservation, edge sharpness, and noise suppression, even in low-dose environments. These distinctions are important criteria for selecting the optimal algorithm and reconstruction level according to the intended use of the image and the purpose of interpretation in future clinical applications. The performance disparities observed between PI and TF techniques may stem from differences in the underlying deep learning network architecture, characteristics of the datasets used for training, and optimization objective functions. The fact that the PI technique offers clearly distinct levels, such as Smoother, Standard, and Sharper, suggests that it is designed to allow users to explicitly control the balance between noise reduction level and sharpness. However, the consistent superiority of the TF technique in GMSD values implies that the algorithm may have been trained in a specialized manner for preserving fine structures and boundary information or that the network architecture itself may be conducive to maintaining these characteristics.

Recently, numerous studies have reported the clinical utility of DLIR technology. For instance, Greffier et al. [31] reported through a phantom study that DLIR algorithms exhibited lower noise and improved structural detection accuracy than conventional IR techniques. Furthermore, Jiang et al. [23] reported that DLIR-TF improved lung nodule detection sensitivity compared to existing ASiR-V and FBP in ultra-low-dose chest CT. Yeom et al. [32] revealed that the emphysema index in ultra-low-dose CT images using DLIR-TF showed no statistically significant difference from that in standard-dose CT. Meanwhile, Philips’ White Paper describes the process by which the PI technique generates images of quality similar to high-dose images from low-dose images through a supervised learning-based convolutional neural network structure, which is consistent with the noise suppression performance of PI-Smoother in this study [21].

The results of this study suggest that DLIR technology, particularly the PI and TF techniques, has the potential to be clinically useful in the field of chest CT. The superior noise reduction capabilities of the PI Smoother and TF techniques can improve image quality in low-dose chest CT screening or follow-up examinations, potentially contributing to an enhanced detection rate of low-contrast lesions, such as ground-glass opacities. Furthermore, the low GMSD values, which represent the fine structure preservation capability demonstrated by the TF technique, may be advantageous for improving the accuracy of evaluating interstitial lung diseases or fine anatomical structures such as small pulmonary nodules and bronchial wall thickness. While prior research has shown that ASIR-V exhibited a significant error by overestimating the volume of 2–6 mm nodules, TF techniques have proven to reduce this error and improve the measurement accuracy of subtle lung nodules. Similarly, the low GMSD values that the TF technique demonstrated in our study give us the expectation that it can more accurately evaluate subtle lung nodules, consistent with the results of previous studies [33].

Conversely, the edge enhancement effect of the PI Sharper level may be helpful in specific situations, such as in clarifying boundaries during the quantitative assessment of bronchiectasis or emphysema. The diagnosis of emphysema is performed through a quantitative evaluation of CT images, which plays a crucial role in patient selection and judging treatment efficacy [34]. Methods for the quantitative assessment of emphysema include a visual scoring method that is susceptible to inter-reader variability, a method that utilizes the number of pixels in the −900 HU to −1000 HU range, and a method of evaluation using the 15th percentile obtained from histogram analysis of lung images. However, when performing a quantitative evaluation of emphysema, a limitation exists with low-dose CT, which is frequently used for screenings [35]. The reduced radiation dose in these images increases noise, which can directly affect the attenuation values of the lung parenchyma. Therefore, it is expected that by utilizing the PI technique to clarify boundaries, these limitations of low-dose CT can be overcome, leading to more accurate results in the quantitative evaluation of emphysema. Ultimately, DLIR technology is expected to play a key role in realizing the ALARA principle and in enhancing patient safety by significantly reducing radiation exposure while maintaining or even improving the image quality necessary for diagnosis.

The relatively low tube voltage of 80 kVp used in this study has important clinical implications. Generally, low-kVp scanning has the advantage of enhancing the image contrast by increasing the X-ray attenuation differences between tissues. This can be particularly effective for maximizing the effect of contrast agents in contrast-enhanced CT examinations. However, low-kVp scanning has notable disadvantages, including increased quantum mottle due to a lower photon count, intensified beam hardening effects, and potential degradation of spatial resolution when using conventional FBP reconstruction methods [18,36]. Nevertheless, advancements in DLIR technology present the possibility of overcoming these limitations of low-kVp, low-dose scanning. As shown in the results of this study, DLIR techniques such as PI and TF can effectively control increased noise under low-kVp conditions. PI Smoother significantly reduced noise, and TF also showed favorable noise characteristics. Furthermore, DLIR is evolving beyond simple noise reduction towards restoring lost signal information and improving spatial resolution. Thus, leveraging the contrast enhancement benefits of low-kVp scanning while mitigating the issues of increased noise and potential resolution degradation through DLIR opens up the possibility of acquiring diagnostically valuable chest CT images at even lower radiation doses.

This study utilized various quantitative image quality assessment metrics such as SNR, PSNR, SSIM, and GMSD. Since a single metric can only reflect specific aspects of image quality, using multifaceted assessment factors is essential for understanding the performance of each DLIR technique from a more comprehensive and balanced perspective. For example, while SNR and PSNR are useful for evaluating noise levels and overall signal fidelity, they do not directly reflect the image’s structural information preservation capability or the sharpness of boundaries. SSIM and GMSD have advantages in evaluating these structural aspects. Therefore, by comprehensively analyzing multiple metrics that reflect such diverse characteristics, one can gain assistance in judging which aspects a specific reconstruction technique excels or falters in, and which metrics should be considered more important depending on the clinical objective. This will ultimately contribute to establishing the most appropriate image reconstruction strategy for specific clinical scenarios.

This study has several limitations that warrant discussion. First, all experiments were conducted exclusively using the AAPM CIRS-610 phantom. While this phantom is a well-established standard for assessing CT image quality and provides consistent, reproducible conditions for algorithm comparison, it inherently lacks the biological and anatomical variability present in actual patients. For example, it cannot mimic the wide range of vascular structures, tissue heterogeneity, and disease manifestations such as subtle calcifications, plaque burden, or irregular lesion margins that may challenge reconstruction algorithms in clinical use. Moreover, phantoms are static, meaning they do not reproduce motion artifacts caused by patient breathing, heartbeat, or involuntary movement, which are frequent in real-world imaging and can significantly affect deep learning reconstruction performance. Consequently, while the phantom allows for controlled, vendor-neutral benchmarking, the findings may not fully capture the complexity of clinical imaging. To ensure the generalizability and clinical validity of these results, further studies using multi-center, patient-based datasets with diverse anatomies, pathologies, and acquisition conditions are essential. In particular, future investigations will be directed toward evaluating whether AI-based reconstruction can enhance the reproducibility of quantitative measurements in low-dose CT examinations of patients with emphysema. The reproducibility of quantitative assessments under low-dose conditions has been recognized as a clinically significant challenge in emphysema management, and the present phantom-based findings provide a rationale for such validation in real patient cohorts. Second, the scope of algorithm comparison in this study was intentionally narrow, focusing only on two DLIR algorithms under a single 80 kVp low-dose acquisition setting. While this targeted design allowed for a controlled head-to-head evaluation, it inevitably limits the generalizability of the findings. DLIR performance may differ substantially when applied to other dose levels (e.g., 100–120 kVp) or under ultra-low-dose protocols, where noise characteristics, photon statistics, and reconstruction behavior change dramatically. Furthermore, we did not include DLIR solutions from other major CT vendors, meaning the results cannot be extrapolated to all commercially available DLIR systems or to future algorithm iterations that may incorporate new architectures or training strategies. In addition, only the manufacturers’ predefined reconstruction presets were examined, including PI Smoother, Standard, and Sharper, and TF Middle and High. These presets reflect commonly used clinical settings but may not represent the full range of reconstruction possibilities. We did not explore intermediate or hybrid settings (e.g., blending between Smoother and Sharper) or fine-tuned parameter adjustments that could allow users to more precisely trade off noise suppression against edge enhancement for different diagnostic needs. Moreover, this study did not provide a systematic failure analysis beyond the evaluation of edge sharpness versus noise suppression. In clinical practice, excessive noise suppression may lead to missed detection of small pulmonary nodules, whereas oversharpening of edges may result in overestimation of calcifications. These potential trade-offs, which could have direct diagnostic consequences, should be specifically addressed in future investigations to provide a more balanced assessment of DLIR performance. Future work should include broader DLIR vendors, multiple kVp ranges, and customizable reconstruction configurations to generate a more comprehensive understanding of how these algorithms perform across diverse imaging environments and clinical requirements. Third, the analysis relied solely on quantitative image quality metrics (SNR, CNR, SSIM, etc.) without incorporating observer studies to determine whether the improvements translate into better diagnostic confidence or accuracy. Because no clinical cases with real pathology (e.g., nodules, microcalcifications, or subtle vascular findings) were included, we could not assess the algorithms’ performance in detecting abnormalities under true diagnostic conditions. Moreover, we did not examine the computational efficiency or workflow feasibility of DLIR in a real-time, clinical environment. Processing time, system integration, and hardware requirements are key considerations for large-scale deployment, particularly in high-volume or emergency settings. Future studies should address these gaps by expanding to multi-center, patient-based research that includes a spectrum of clinical pathologies, additional DLIR vendors and settings, and multiple dose protocols. Observer-based reader studies should complement quantitative metrics to evaluate diagnostic impact, and assessments of computational performance will be necessary to ensure that DLIR solutions can be deployed effectively in clinical workflows. These steps will help move DLIR from controlled phantom experiments toward robust, clinically proven solutions for low-dose CT imaging. In addition, this study relied exclusively on descriptive statistics for reporting the results. Because phantom images do not exhibit the meaningful variability typically encountered in patient datasets, inferential statistical tests could not be applied. This reliance on descriptive comparisons represents a limitation, and future studies incorporating patient-based data with inherent variability will be required to enable robust statistical testing and more comprehensive validation. Finally, while this study referred to potential clinical implications, these should be regarded as preliminary and hypothesis-generating. The findings point to possible directions for future validation studies, particularly those involving patient datasets and observer-based diagnostic evaluations.

## 5. Conclusions

This study conducted a detailed comparative evaluation of the image quality characteristics between two commercial deep-learning-based CT image reconstruction techniques, PI and TF, using an AAPM phantom under 80 kVp low-dose conditions with various quantitative metrics to elucidate their differences. The results indicated that the TF algorithm, particularly the TF-H level, generally demonstrated superior performance in most phantoms compared to the PI algorithm, exhibiting excellent noise suppression capabilities (SNR, nRMSE), structural fidelity (SSIM, FSIM, UQI), and low structural distortion (GMSD). Regarding edge sharpness (Gradient), the TF-M outperformed PI-Sharper in linearity and artifact modules, whereas PI-Sharper exhibited better edge delineation in the high-resolution module. These findings suggest that PI and TF algorithms each possess unique image reconstruction characteristics and that no single DLIR algorithm demonstrates absolute superiority across all evaluation metrics. Therefore, to obtain optimal image quality in clinical settings, selecting the appropriate DLIR algorithm and reconstruction level is crucial and should be guided by considering the diagnostic purpose and key evaluation criteria, such as fine-structure analysis, lesion boundary conspicuity, or overall noise level.

## Figures and Tables

**Table 1:** Computed Tomography (CT) scanning parameters used in the phantom study for both Philips CT 5300 and GE Revolution scanners, detailing Filtered Back Projection (FBP) and Low-dose protocols.

	Phantom Study			
	Philips CT 5300		GE Revolution	
	FBP	Lowdose	FBP	Lowdose
kVp	120	80, 100, 120	120	80
Eff. mAs	33	19, 11, 6	80	45
Pitch	0.8	0.8	0.992:1	0.992:1
Scan time	7.8 s	7.8 s	3.23 s	3.23 s
Rotation time	0.5 s	0.5 s	0.5 s	0.5 s
Slice thickness	1 mm	1 mm	1 mm	1 mm
Increment	1 mm	1 mm	1 mm	1 mm
Scan mode	Helical	Helical	Helical	Helical
Direction	Craniocaudal	Craniocaudal	Craniocaudal	Craniocaudal
Image reconstruction	-	Smoother, Standard, Sharper	-	Middle, High

## Data Availability

The data presented in this study are provided at the request of the corresponding author, requiring the necessary permission to provide the source data from the manufacturer.
